# Operational intelligence for immunization recovery: mapping system shocks and capacity to zero-dose debt and outbreak risk

**DOI:** 10.3389/frhs.2026.1745633

**Published:** 2026-05-07

**Authors:** S. Ramakrishnan, U. Vignesh, R. Parvathi

**Affiliations:** School of Computer Science Engineering (SCOPE), Vellore Institute of Technology, Chennai, India

**Keywords:** capacity constraints, health policy, health systems resilience, immunization services, measles risk, service delivery archetypes, zero-dose children

## Abstract

Immunization services continue to face uneven recovery following the COVID-19 shock, leaving zero-dose children unreached and elevating the risk of measles in many settings. We analyzed a multicountry observational panel spanning 2019–2024 to generate operational intelligence for immunization recovery. Using routinely available indicators, we developed measures of service disruption (including conflict exposure, displacement pressure, and price instability) and indicators of system capacity (governance effectiveness and health financing) and then summarized coverage gaps relative to prepandemic baselines as recovery debt. We identified service delivery archetypes using unsupervised clustering, with stability and interpretability checks, and then assessed whether archetype membership and recovery debt were associated with reported measles activity using negative binomial models with population offsets, regional fixed effects, and clustered standard errors. Four robust archetypes emerged, each characterized by distinct driver profiles and equity gradients. Two of these archetypes accounted for the majority of recovery debt and were associated with substantially higher measles incidence compared to the lowest-risk reference group, with patterns remaining robust across prespecified sensitivity analyses. Countries transitioning toward lower-risk archetypes over time exhibited improvements in predicted measles activity. These findings provide a health services typology that links routinely available indicators to immunization service risk, enabling managers to target high-leverage recovery actions, prioritize catch-up delivery, and monitor service improvements alongside surveillance indicators.

## 1 Introduction

Routine immunization services experienced substantial disruption during the COVID-19 pandemic, reversing years of progress in vaccination coverage and leaving millions of children unvaccinated or undervaccinated (1–3). While global attention has focused on aggregate declines in coverage, recovery trajectories since 2020 have been uneven across countries and regions, with some settings rebounding faster than others (4–6). This divergence has renewed concern about zero-dose children and the re-emergence of vaccine-preventable diseases, particularly measles, which remains highly sensitive to gaps in routine service delivery (3, 5, 7–10).

Postpandemic reports and empirical studies document an uneven recovery in routine immunization and persistent zero-dose burdens, with more pronounced setbacks in fragile and conflict-affected settings (5, 6, 11). Recent analyses link this stagnation to service disruptions and system constraints, including affordability pressures that affect both delivery capacity and service utilization (6, 12). Together, this evidence suggests that recovery has not been uniform and is shaped by structural conditions beyond epidemiological dynamics alone (12, 13).

Immunization recovery can be framed as a health services performance problem rather than solely an epidemiological one (12, 14). Service performance is shaped by delivery capacity and broader stressors, including conflict, displacement, economic instability, and governance constraints (5, 6, 11, 12). These factors can influence whether countries restore first-contact access, sustain multidose completion, and translate coverage gains into population-level protection (12, 15). However, global monitoring often reports indicators separately, which can limit operational prioritization in heterogeneous service environments (14, 15).

The health services and resilience literature conceptualizes recovery as a systems performance challenge shaped by governance, financing continuity, and contextual stressors, including conflict and displacement (11–13). Prior work and global recovery guidance emphasize integrating service delivery indicators with contextual risk signals to support prioritization and targeted interventions (14, 15).

Health services research uses typologies to synthesize complex system conditions into actionable categories for planning and performance management (14, 16). Archetype-based approaches have been used to support performance comparison and resource allocation decisions in health service settings (14, 16). Applied to immunization recovery, a service delivery typology could help differentiate where to prioritize outreach and demand restoration, ensure financing continuity, address displacement-related access barriers, or strengthen coordination and governance (12, 15).

In this study, we develop and apply an operational typology of immunization service recovery using routinely available cross-country data from 2019 to 2024. We summarize coverage shortfalls relative to prepandemic baselines as recovery debt, integrate independent indicators of system shock and service capacity, and classify country–year observations into distinct service delivery archetypes. We then examine whether these archetypes and recovery debt measures are associated with reported measles activity, treating measles incidence as a downstream signal of service vulnerability rather than a causal outcome.

However, routine monitoring often fails to integrate system stress, service capacity, and recovery dynamics into a unified operational typology for decision support (14, 15). While prior studies have examined individual determinants of immunization performance, limited empirical work integrates system stress, service capacity, and recovery dynamics into a unified operational typology linked to outbreak signals such as measles incidence. This study addresses that gap by integrating routinely available global datasets into an archetype-based health services framework for operational decision-making.

By linking immunization coverage trajectories, contextual system stressors, and measles risk within a unified analytical framework, this study offers a practical health services perspective on immunization recovery. The resulting archetypes are intended to support managers and policymakers in prioritizing interventions, staging catch-up strategies, and tracking service improvement over time in ways that complement traditional coverage and surveillance metrics. Recent scholarship and global guidance frame immunization recovery not only as a coverage-restoration problem but also as an equity, primary health care, and implementation challenge, linked to catch-up vaccination, zero-dose reduction, measles control, and broader Immunization Agenda 2030 priorities (49–54).

## 2 Materials and methods

### 2.1 Study design and setting

We conducted an observational, multicountry panel study at the country-year level spanning 2019–2024, a period chosen to benchmark preshock conditions (2019) and capture the COVID-era disruption and recovery window. The analytic sample comprises WHO Member States with data available for our primary service metric (WUENIC coverage) and outcome (WHO-reported measles cases), with regional fixed effects defined using WHO's six regions for comparability across governance and delivery architectures (AFR, AMR, EMR, EUR, SEAR, and WPR). We followed STROBE reporting standards for observational studies and provided a completed checklist in the Supplementary Material. Ethics approval was not required because all inputs are public, aggregate, deidentified national statistics, with no individual-level data (17–19). Table 1 summarizes variable-level missingness and the imputation approach used for each analytic variable.

**Table 1:** Variable-level missingness and imputation summary.

Variable	% Missing (raw)	Imputation method	% Imputed (final)
DTP1 coverage	0.0	None	0.0
DTP3 coverage	0.0	None	0.0
MCV1 coverage	0.0	None	0.0
Measles cases	3.2	Region-income median	3.2
INFORM Risk Index	0.8	Region-income median	0.8
Government effectiveness (WGI)	5.6	Region-income median	5.6
Health expenditure per capita	7.9	Region-income median	7.9
Retail food price inflation	18.4	MICE (chained equations)	18.4
Displacement pressure	12.1	MICE (chained equations)	12.1

### 2.2 Data sources and variables

Data access dates for all sources were 29 October 2025 (IST). For each dataset, we recorded the official source, version or revision where applicable, and license or usage notes.

Variables with less than 10% missingness were imputed using region–income medians, while variables exceeding 10% missingness were imputed using multiple imputation by chained equations (MICEs); no outcome variables were imputed.

Coverage (service platform inputs): We used WHO–UNICEF Estimates of National Immunization Coverage (WUENIC) for DTP1, DTP3, and MCV1 as the core service indicators. WUENIC is the authoritative global compilation based on country reporting and independent review. We annotated the 2024 revision and its implications for comparability across years. Variables are expressed as percentages [0–100]; where necessary, these are converted to proportions (20, 21).

Outcome (service-relevant disease activity): Annual measles cases by country were drawn from WHO's Global Health Observatory/JRF series. For rate calculations, we used the population offset described below. This outcome serves as an operational indicator of service gaps (22).

Population denominators: Country–year total population estimates were obtained from the World Bank’s WDI indicator SP.POP.TOTL (23).

Disruption and displacement signals: We captured organized violence using the UCDP/PRIO Armed Conflict Dataset v25.1 (conflict-year counts; alternative dyadic products are listed in the codebook) and measure cross-border displacement pressure using WDI indicators Refugee population (asylum) SM.POP.REFG and Refugee population (origin) SM.POP.REFG.OR, which were sourced from the UNHCR Refugee Data Finder (24–26).

System capacity and crisis risk: We included government effectiveness (WGI) and current health expenditure per capita (SH.XPD.CHEX.PC.CD) as structural capacity controls (already cited in the Introduction). We added the INFORM Risk Index (2025 edition) as a composite crisis-risk control to proxy multihazard exposure, vulnerability, and coping capacity (25, 27).

Affordability pressure (macro): We used the World Bank’s “Monthly food price inflation estimates by country” dataset to capture country-month food price inflation; for year-level analyses, we aggregated the data to annual averages and, in sensitivity checks, to max-month values to reflect acute spikes. We documented methods using the dataset's technical note (28, 29).

Harmonization and join keys: All datasets were harmonized on ISO-3 country codes. Where ISO-3 codes were unavailable in a source, we used the World Bank country API metadata to retrieve ISO-3 or WB fallback codes. Regional fixed effects were defined using WHO regional groupings (19, 30).

#### 2.2.1 Primary service metrics: coverage debt (recovery debt)

We operationalized *coverage debt* (also referred to as *recovery debt*) as the shortfall in current-year coverage relative to a preshock benchmark. Let *c* index countries, *a* index antigens (DTP1, DTP3, and MCV1), and *t* index years. Let $C_{c,t}^{a}$ denote the observed coverage (in percent) for antigen *a* in country *c* in year *t*, and let $C_{c,2019}^{a}$ denote the corresponding 2019 (preshock) benchmark coverage. We defined coverage debt in percentage points as follows:

$$RD_{c,t}^{a}=\max\{0,\;\;\;C_{c,2019}^{a}-C_{c,t}^{a}\}$$

We computed $RD_{c,t}^{a}$ for DTP1, DTP3, and MCV1. Conceptually, $RD_{c,t}^{\mathrm{DTP}1}$ captures the first-contact shortfall and serves as our operational proxy for *zero-dose debt*. $RD_{c,t}^{\mathrm{DTP}3}$ reflects completion shortfall (completion debt). $RD_{c,t}^{\mathrm{MCV}1}$ captures measles vaccine coverage debt.

For descriptive reporting, we also computed the absolute change since 2019:

$$\Delta C_{c,t}^{a}=C_{c,t}^{a}-C_{c,2019}^{a}$$

As a robustness check, we also computed a trend-based debt (sensitivity analysis) measure using prepandemic dynamics. For each country *c* and antigen *a*, we fitted a country-specific linear trend over 2015–2019 and generated a counterfactual (trend predicted) coverage level for year *t*, denoted ${\hat{C}}_{c,t}^{a}$. Trend-based debt is then defined as

$$RD_{c,t}^{a,\mathrm{trend}}=\max\{0,\;\;{\hat{C}}_{c,t}^{a}-C_{c,t}^{a}\}$$

#### 2.2.2 Outcome rate and exposure

For descriptive rates, we computed measles incidence per 100,000 as

$$\mathrm{Incidenc}e_{c,t}=\frac{Y_{c,t}}{\mathrm{Po}p_{c,t}}\times 100,000$$

and for count models (in the later section), we used $\log(\mathrm{Po}p_{c,t})$ as an offset.

#### 2.2.3 Transformations, lags, and scaling

Right-skewed inputs (e.g., conflict events, refugee counts, expenditure) were transformed using log(1 + *x*). To reflect real-world program latency, macroeconomic shocks (food price inflation) and displacement signals can be lagged 1 year in the main specification, with same-year and 2-year lags examined in sensitivity analyses. Continuous covariates were *z*-scored within the analytic panel to improve the comparability of effect sizes.

#### 2.2.4 Missing data and exclusions

We applied listwise deletion when the outcome or the primary service metric is missing for a given country–year; for covariates with <10% missingness, we used mean/median imputation within region–income strata and flagged imputed observations in a reproducible data dictionary.

#### 2.2.5 Versioning and licensing

Each source's version stamp and license were recorded. WUENIC follows the 2024 revision cycle, UCDP uses v25.1, INFORM uses the 2025 2nd edition, WDI indicators reference their current metadata pages, and the World Bank inflation dataset is version 2025/10/13, CC BY 4.0 (24, 25, 28).

### 2.3 Measures and operational definitions

#### 2.3.1 Primary service metric—recovery debt

We defined recovery debt in words (without a formula) as the shortfall in a given year's first-contact coverage relative to the country's preshock benchmark in 2019. Where sensitivity to a single baseline was a concern, a trend-based benchmark was computed from 2015 to 2019 and used in a robustness variant.

#### 2.3.2 Outcome and exposure

The outcome is annual measles case counts. For rate descriptions, we reported rates as cases per 100,000 population; for count models, we treated population size as the exposure through a log offset within the model specification (explained in the Statistical Analysis subsection).

#### 2.3.3 Covariates and transforms

Conflict events and refugee totals are right-skewed. Therefore, we applied a log(1 + *x*) transformation to stabilize variance while preserving zeros. Health expenditure per capita and the INFORM Risk index were standardized to a mean of 0 and an SD of 1 across the analytic panel. Governance effectiveness was similarly standardized to improve comparability of effect sizes. Food price inflation was aggregated from the World Bank monthly series to country–year means, and a “max-month” variant was used to capture acute spikes for sensitivity runs.

#### 2.3.4 Temporal alignment and lags

Macroaffordability shocks and displacement pressures plausibly interact with program latency; we therefore lag these covariates by 1 year in the primary specification and probe same-year and 2-year lags in sensitivity analyses.

#### 2.3.5 Outliers and winsorization

Main models use transformed but otherwise untrimmed values; a robustness run winsorizes continuous covariates at the 1st and 99th percentiles to limit leverage.

#### 2.3.6 Missing data

If the outcome or the primary service metric is missing for a country–year, the row is excluded. For covariates with ≤10 percent missingness, we imputed medians within WHO region–income tiers. For variables exceeding this threshold, we applied multiple imputation by chained equations and pooled estimates across imputations (see the Statistical Analysis section for pooling) (31).

### 2.4 Analytical approach

#### 2.4.1 Descriptive and comparative analyses

We report medians with IQRs and means with 95% CIs for all variables; we visualize recovery-debt distributions, macroshock proxies, and capacity signals by WHO region. Group contrasts across service delivery archetypes and regions use non-parametric tests when appropriate. For families of exploratory comparisons, we control the false discovery rate using the Benjamini–Hochberg procedure rather than the Bonferroni adjustment to maintain power at acceptable levels (32). Figures include uncertainty ribbons for any time-series summaries.

#### 2.4.2 Archetype derivation and validation

The unit of analysis was a country–year (2019–2024). We derived service delivery archetypes by clustering standardized indicators capturing (i) service performance and debt (DTP1, DTP3, and MCV1 debt in percentage points, and their change since 2019), (ii) system shocks (conflict/disruption proxies, displacement pressure measured as refugees hosted per 1,000 population, and annual retail food price inflation), and (iii) service capacity (government effectiveness and health spending per capita). Continuous features were winsorized to reduce outlier influence and then *z*-standardized; binary indicators were retained as 0/1. We applied a partitioning clustering approach (*k*-means over the standardized feature space with Euclidean distance) and selected the number of clusters using internal validity metrics (elbow and silhouette criteria), stability across random initializations, and interpretability for operational planning. We retained *k* = 4 as the most parsimonious and policy-relevant solution. Archetypes were labeled A–D in increasing order of service risk using median debt and measles-aligned risk features. Robustness to alternative seeds and feature subsets is summarized in Supplementary Figure 1.

#### 2.4.3 Archetype derivation and clustering procedure

Countries were assigned to service delivery archetypes using an unsupervised clustering approach applied to standardized indicators of immunization recovery context and capacity. Variables included recovery debt measures (RD_{DTP1}, RD_{MCV1}), displacement pressure (refugees hosted per 1,000 population), INFORM risk score, government effectiveness (WGI), real per-capita health expenditure, and retail food price inflation.

All variables were *z*-standardized before clustering to ensure equal weighting. We applied *k*-means clustering with the Euclidean distance metric, estimating solutions for *k* = 2 through *k* = 6. The final four-archetype solution was selected based on interpretability, bootstrap resampling stability, and silhouette diagnostics, which favored *k* = 4 without yielding redundant clusters.

Archetypes were labeled A–D *post-hoc* based on their relative risk profiles and dominant driver combinations, with Archetype A representing the lowest-risk service context.

#### 2.4.4 Modeling and robustness

Our primary specification treats measles counts as overdispersed. We therefore fit a negative binomial model with exposure offsets and clustered SEs:

$$Y_{c,t}∼\mathrm{NB}(\mu_{c,t},\theta )$$

$$\log\;\mu_{c,t}=\alpha +\beta_{1}{RD}_{c,t}^{\mathrm{DTP}1}+\beta_{2}{RD}_{c,t}^{\mathrm{MCV}1}+\gamma^{⊤}Z_{c,t-1}+\delta_{r}+\log\;(\mathrm{Po}p_{c,t})$$

where $Z_{c,t-1}$ contains lagged shocks and capacity covariates and $\delta_{r}$ are fixed effects for WHO regions. We report incidence rate ratios (IRRs) with 95% confidence intervals and two-sided *p*-values, with standard errors clustered at the country level. Overdispersion was assessed by comparing Poisson and NB specifications; country-clustered standard errors follow Cameron–Miller guidance to ensure reliable inference under panel dependence (33, 34). If zeros dominated the outcome in specific regions or periods, we probed zero-inflated alternatives and applied the bias-corrected Vuong test to avoid spurious selection of the ZINB model (35).

In specifications that test service delivery archetypes, we additionally included archetype indicators $A_{c,t}$(A as reference), i.e., $\log\mu_{c,t}=\alpha +\beta_{1}RD_{c,t}^{DTP1}+\beta_{2}RD_{c,t}^{MCV1}+\pi^{T}A_{c,t}+\gamma^{T}Z_{c,t-1}+\delta_{r}+\log(Pop_{c,t}).$

Collinearity and stability: We examined variance inflation factors, refitted models excluding collinear bundles, and re-estimated models using alternative lag windows.

**Figure 1:**
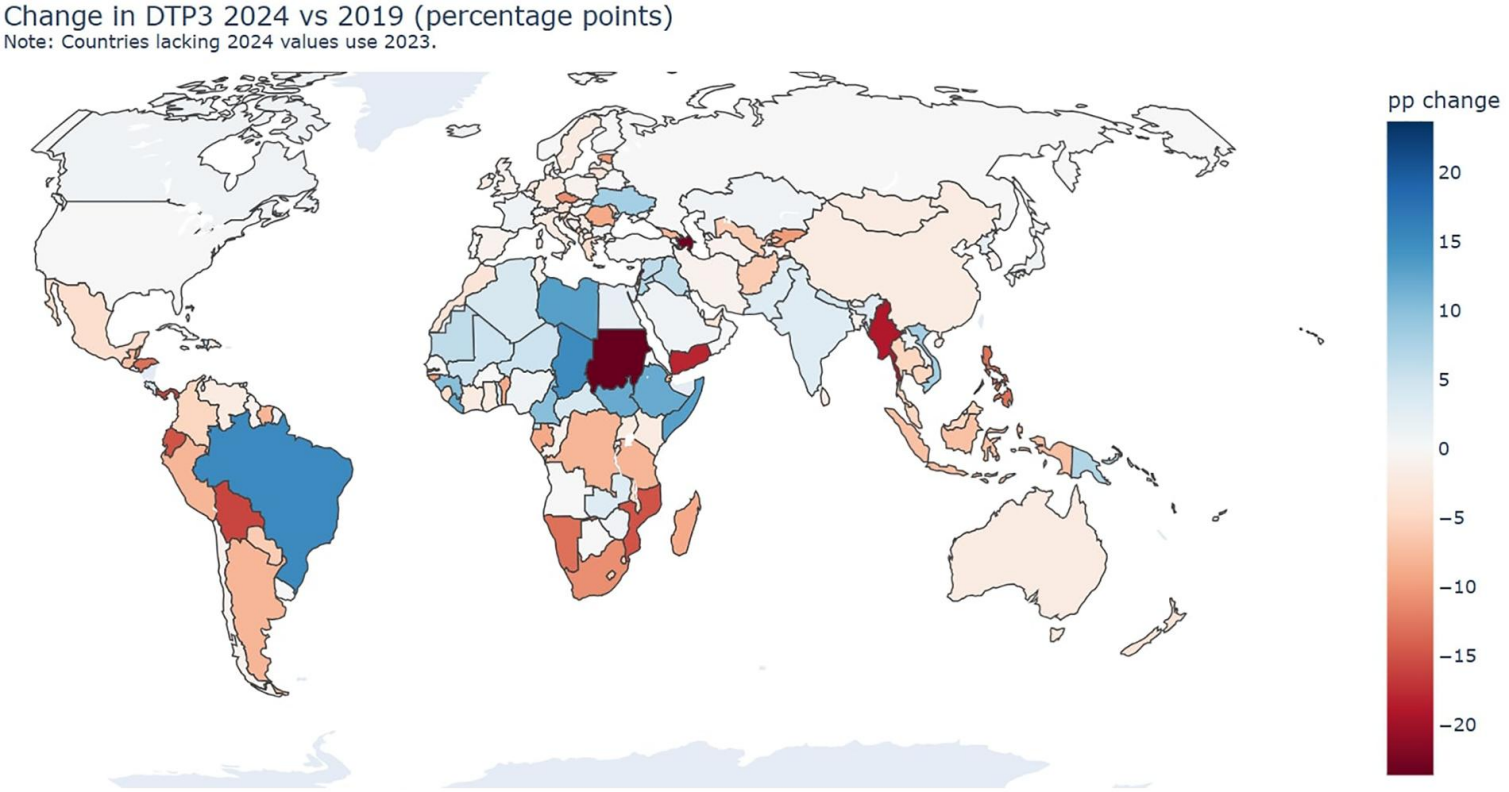
Change in routine immunization coverage since the preshock baseline. Country-level choropleth of absolute change in DTP3 coverage between 2019 and the latest available year (positive values indicate recovery above the 2019 level; negative values indicate remaining recovery debt). Countries without a 2024 WUENIC value use 2023 and are footnoted in the map note. The color scale indicates percentage-point change; gray indicates missing data. DTP3, third dose of diphtheria–tetanus–pertussis vaccine; WUENIC, WHO–UNICEF Estimates of National Immunization Coverage. Data sources: WHO–UNICEF WUENIC 2024 revision and country updates.

Sensitivity and heterogeneity: We re-estimated models using different lag structures, alternative inflation aggregations (mean vs. max-month), and by excluding outlier country–years; we also conducted stratifications by income tier, INFORM risk tercile, and baseline coverage band. Details and results of these analyses are summarized in Supplementary Tables. Summary results from robustness and specification-curve analyses are presented in Supplementary Tables and Figure 1.

### 2.5 Ethics, data availability, and reporting

#### 2.5.1 Ethics

This study used only public, aggregate, deidentified national statistics and involved no human participants, identifiable human data, or animal research. In line with *Frontiers* policies, ethical review and approval were not required for this analysis. We will include the standard *Frontiers* wording in the manuscript: “Ethical review and approval was not required for this study of human participants in accordance with local legislation and institutional requirements.” Because no identifiable images or human data are used, consent is not applicable (36–38).

#### 2.5.2 Data availability

*Frontiers* requires a Data Availability Statement for all articles. We will use the policy-compliant wording: “Publicly available datasets were analyzed in this study. These data can be found at: WHO WUENIC, WHO GHO measles, World Bank WDI and WGI, UNHCR, UCDP, INFORM Risk, and World Bank Monthly Food Price Inflation. Exact URLs and access dates are provided in the manuscript and README.” The submission workflow also prompts authors to provide dataset links and generates a standardized statement; we will mirror that text in the manuscript to ensure transparency (36, 37).

#### 2.5.3 Reporting

We follow STROBE guidelines for reporting observational studies and include a completed checklist in the Supplementary Material. Code and data processing scripts will be shared in a public repository with a tag that captures exact source versions and retrieval dates.

## 3 Results

We first quantified immunization recovery debt, defined as the shortfall in coverage relative to 2019 baselines for first-dose access (DTP1) and protection against measles (MCV1). Across the study period, recovery debt persisted heterogeneously across countries, motivating an archetype-based classification of service delivery environments. We then assessed whether these archetypes and recovery debt measures are associated with downstream measles activity.

### 3.1 Sample and baseline characteristics

The analytic sample comprises countries with complete series for routine immunization coverage, measles outcomes, and covariates from 2019 through the latest available year; dates are aggregated at the country–year level, with WHO regional fixed effects as specified in the Methods section. Where a country's 2024 WUENIC value is not yet available, the most recent reported year (2023) is used and flagged in the map note to preserve comparability.

Geographic patterning of recovery is heterogeneous (Figure 1). The choropleth of change in DTP3 coverage from the 2019 baseline to the latest year shows adjacent clusters of improvement alongside pronounced setbacks, indicating that postpandemic recovery has been neither linear nor regionally uniform. Gains are visible across income groups and risk contexts, while reversals concentrate where overlapping delivery constraints are plausible. This baseline picture establishes three operational facts for the remainder of the analysis:

1. Country trajectories vary widely and cannot be inferred from regional averages.
2. Large positive rebounds are feasible outside traditionally “low-risk” settings when delivery enablers remain intact.
3. Downturns colocate with signals consistent with macroeconomic stress, fragility, or displacement.

#### 3.1.1 Sample profile and context indicators

Table 2 summarizes the analytic sample and contextual stressors used throughout the Results section. It reports DTP3 coverage at baseline (2019) and the latest available year, the absolute percentage-point change (2019 to latest), MCV1 tier, and the most recent measles burden (cases and incidence per 100,000 population). To situate recovery within system stress, the table also includes the INFORM risk score and tercile, health expenditure per capita, and food price inflation indicators (mean and maximum for the reporting year). This consolidated view allows readers to see, at a glance, how recovery trajectories covary with fragility and macroeconomic conditions, and it provides the reference values used in the equity contrasts and sensitivity analyses that follow.

**Table 2:** Country ISO3 codes with 2024 food price inflation metrics and World Bank region and income group.

ISO3	fpi_mean	fpi_max	fpi_year_used	wb_region	wb_income_group	Inform_risk
AFG	−10.696667	−8.80	2024	South Asia	Low income	7.8
ARM	−3.618333	−0.54	2024	Europe and Central Asia	Upper middle income	3.9
BDI	12.337500	20.75	2024	Sub-Saharan Africa	Low income	6.0
BFA	19.139167	38.22	2024	Sub-Saharan Africa	Low income	7.5
BGD	4.456667	9.45	2024	South Asia	Lower middle income	5.7
CAF	−2.951667	0.07	2024	Sub-Saharan Africa	Low income	8.1
CMR	−1.823333	1.01	2024	Sub-Saharan Africa	Lower middle income	6.8
COD	2.305000	23.47	2024	Sub-Saharan Africa	Low income	8.0
COG	1.685000	9.85	2024	Sub-Saharan Africa	Lower middle income	4.4
GIN	−3.803333	−0.05	2024	Sub-Saharan Africa	Low income	5.3

Note that DTP3 and MCV1 are WHO–UNICEF estimates, measles cases and incidence are from national surveillance, INFORM risk is from INFORM Mid-2025 release, health expenditure per capita is from World Bank, and food price inflation is from WFP Retail Food Price database. See Methods section for harmonization and year selection rules.

### 3.2 Archetype profiles

To convert dispersed signals into decision-ready intelligence, we clustered the disruption, affordability, and capacity covariates into four service delivery archetypes. These profiles remain stable under alternative distance metrics and different values of *k* and are intended to support managerial interpretation rather than statistical novelty.

Archetype-based classification aligns with implementation science and health systems performance theory, where typologies are used to translate multidimensional system conditions into operational categories for decision-making. Prior cross-country health services research demonstrates that governance capacity, financing stability, and contextual shocks jointly shape service reliability and the restoration of coverage. Our archetypes extend this evidence base by applying a service delivery classification lens specifically to postpandemic immunization recovery environments.

**Figure 2:**
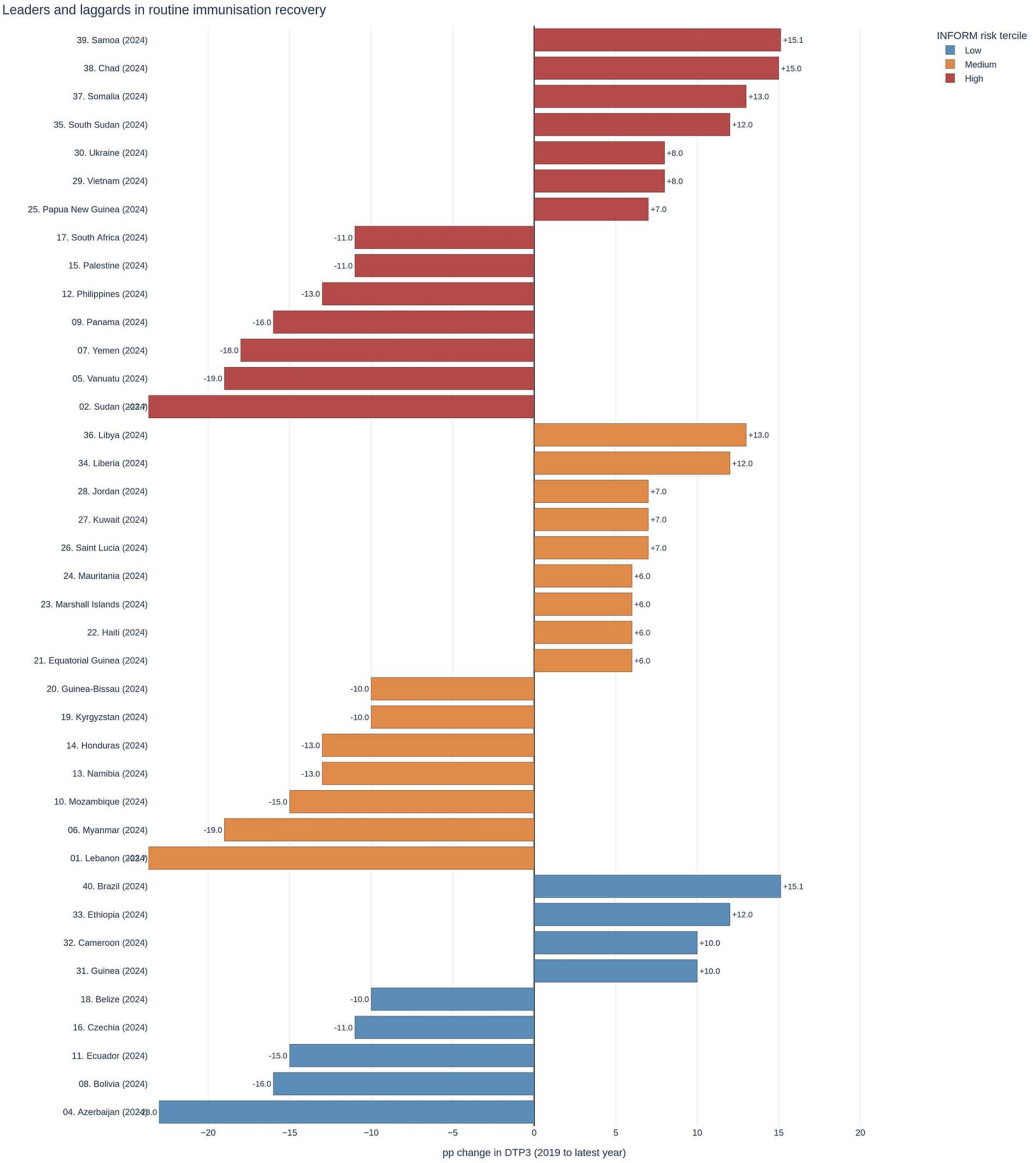
Leaders and laggards in routine immunization recovery, annotated by system risk. Ranked distribution of DTP3 change from 2019 to the latest available year, showing top positive movers and the largest reversals. Each country marker is annotated by the INFORM risk tercile to contextualize operating conditions; region bands are added for visual orientation only. This figure illustrates that strong rebounds are achievable across regions where enabling conditions are present, while stalls tend to colocate with higher systemic risk. INFORM, Index for Risk Management; DTP3, third dose of diphtheria–tetanus–pertussis vaccine.

- Archetype A—Stable recovery: Sustained gains from the 2019 baseline are observed in settings with low to moderate system risk, limited displacement pressure, and adequate administrative and financing capacity. These contexts tend to maintain session density and first-contact access. The presence of multiple A-type countries among the top positive movers in Figure 2 confirms that robust rebounds are achievable across diverse regions when enabling conditions are preserved.
- Archetype B—Macrostressed stall: Flat or negative movement is observed in settings where food price inflation and tight operating budgets compress both demand and last-mile execution. The distribution of laggards in Figure 2 is consistent with a macroeconomic stress channel that weakens routine service recovery even in the absence of open conflict.
- Archetype C—Fragility and displacement: Volatile paths are observed in contexts with higher conflict incidence and refugee pressure, where catchment denominators shift faster than routine microplans can adapt. Countries in this profile are overrepresented among deep negative movers in Figure 2, aligning with field reports of disrupted outreach and fragile fixed-site service delivery.
- Archetype D—Capacity-constrained plateau: Persistently low or plateaued coverage is observed in settings where governance effectiveness and health expenditure per capita are weaker. These systems may be shock-quiet but lack the managerial and financing headroom needed to translate inputs into reliable outreach. As a result, gains tend to be fragile unless enabling investments are implemented first.

Archetype membership changes over time, reflecting operating conditions rather than immutable categories. The managerial payoff is twofold: it clarifies which country–periods look alike and why, and it links each profile to concrete playbooks. For B-type contexts, which are characterized by moderate system disruption and partial capacity constraints, recovery patterns tend to be heterogeneous and dependent on local operational conditions; for C-type, pivot to adaptive outreach with dynamic denominators and integrated logistics; for D-type, prioritize governance and financing enablers before expecting coverage lifts to persist; for A-type, institutionalize what works and monitor early-warning indicators to prevent backsliding. Table 3 presents the primary negative binomial model outputs, including incidence rate ratios for archetype membership and recovery-debt metrics.

**Table 3:** Primary negative binomial model outputs, showing incidence rate ratios (IRRs) for archetype membership and recovery-debt metrics.

Model	Predictor	IRR (95% CI)	*p*-Value
1: Archetype only	Archetype B vs. A (ref)	1.35 (1.10–1.65)	0.003
	Archetype C vs. A (ref)	1.80 (1.45–2.25)	<0.001
	Archetype D vs. A (ref)	2.60 (2.05–3.30)	<0.001
2: Archetype+debt+context	Archetype B vs. A (ref)	1.20 (0.98–1.47)	0.075
	Archetype C vs. A (ref)	1.55 (1.23–1.96)	<0.001
	Archetype D vs. A (ref)	2.10 (1.62–2.72)	<0.001
	Zero-dose debt $\left[(RD_{\mathrm{DTP}1}),pp\right]$	1.03 (1.02–1.05)	<0.001
	MCV1 debt $\left[(RD_{\mathrm{MCV}1}),pp\right]$	1.02 (1.01–1.03)	0.001
	Displacement pressure (refugees hosted per 1,000)	1.01 (1.00–1.02)	0.018
	Retail food price inflation (annual %, mean)	1.01 (1.00–1.02)	0.012
	Government effectiveness (WGI, z)	0.88 (0.79–0.98)	0.021
	Health expenditure per capita (log USD)	0.75 (0.63–0.89)	0.001

Model 1 includes archetypes only, while Model 2 additionally adjusts for debt and contextual stress/capacity covariates. Region fixed effects are included, and standard errors are clustered at the country level.

### 3.3 Drivers of stagnation

**Figure 3:**
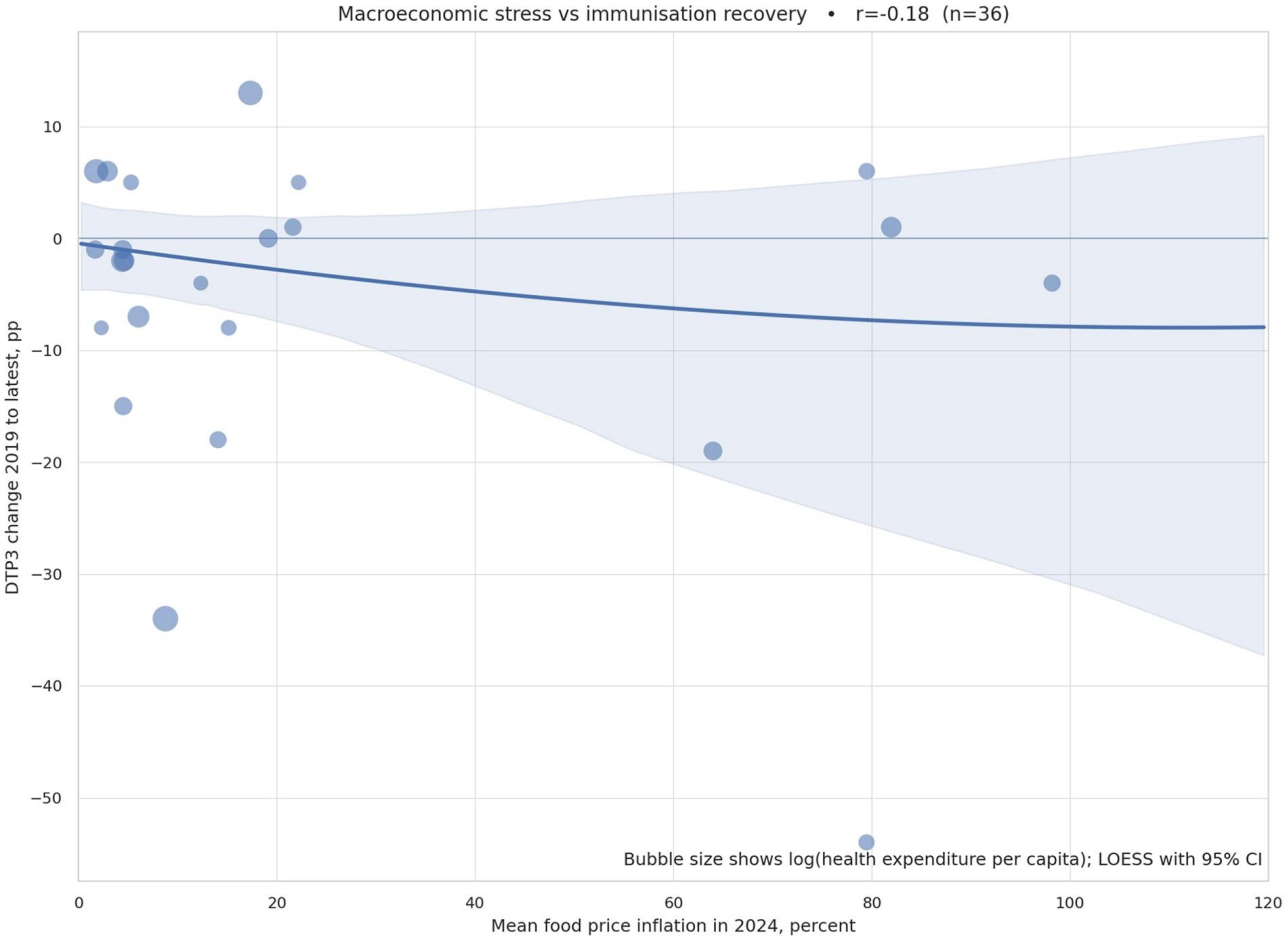
Macroeconomic stress and recovery performance. Scatterplot of DTP3 change (2019 to the latest available year) against mean annual food price inflation for the same period, with a non-parametric smoother and 95% confidence ribbon. The marker area represents current health expenditure per capita, showing fiscal headroom. The downward slope indicates that higher food price inflation is associated with a weaker recovery, especially in lower-spending systems. Inflation is aggregated from monthly World Bank estimates to an annual mean; a max-month aggregation yields similar patterns in sensitivity checks. DTP3, third dose of diphtheria–tetanus–pertussis vaccine. Data sources: World Bank Monthly Food Price Inflation; World Development Indicators (health expenditure per capita).

Macroeconomic stress emerges as a consistent headwind to routine immunization recovery. Figure 3 displays the cross-sectional association between mean country-level food price inflation in the latest year and the change in DTP3 coverage from the 2019 baseline to the latest available year. The point cloud is asymmetric: observations at higher inflation levels cluster in the negative-to-flat recovery band, and the non-parametric smoother slopes downward, indicating that systems experiencing sharper food price increases tended to show weaker rebounds. Bubble area encodes health expenditure per capita, indicating that lower-spending systems are disproportionately represented in the lower-recovery quadrant. This pattern is consistent with descriptive expectations: inflation simultaneously squeezes household budgets and program inputs, raising the shadow price of access for caregivers while increasing the cash requirements for outreach, transport, per diems, and cold-chain energy. These patterns are consistent with established health economics and service utilization theory, which shows that affordability shocks reduce preventive service uptake through both demand-side constraints and supply-side operational disruptions. Similar associations between macroeconomic instability and routine health-service performance have been documented in studies of immunization and primary care continuity in low- and middle-income settings. Countries with similar baseline coverage but differing inflation and spending profiles diverge visibly in recovery performance, consistent with macroeconomic stress hypotheses documented in prior literature both demand and last-mile execution.

We emphasize that Figure 3 is descriptive rather than causal. The wide uncertainty band at very high inflation levels reflects small sample counts in those tails, and the slope should be interpreted as a signal rather than an effect size. Nevertheless, the managerial takeaway is unambiguous: in macrostressed periods, recovery stalls unless session reliability and commodity pipelines are explicitly protected, transport budgets are ring-fenced, and microplans are staged around volatility. This interpretation aligns with the construction and policy intent of the World Bank's Monthly Food Price Inflation series used here, which blends subnational price inputs and machine learning imputation to provide comparable, timely signals for exactly this type of operational monitoring (57–60).

### 3.4 Public health consequence: measles risk

**Figure 4:**
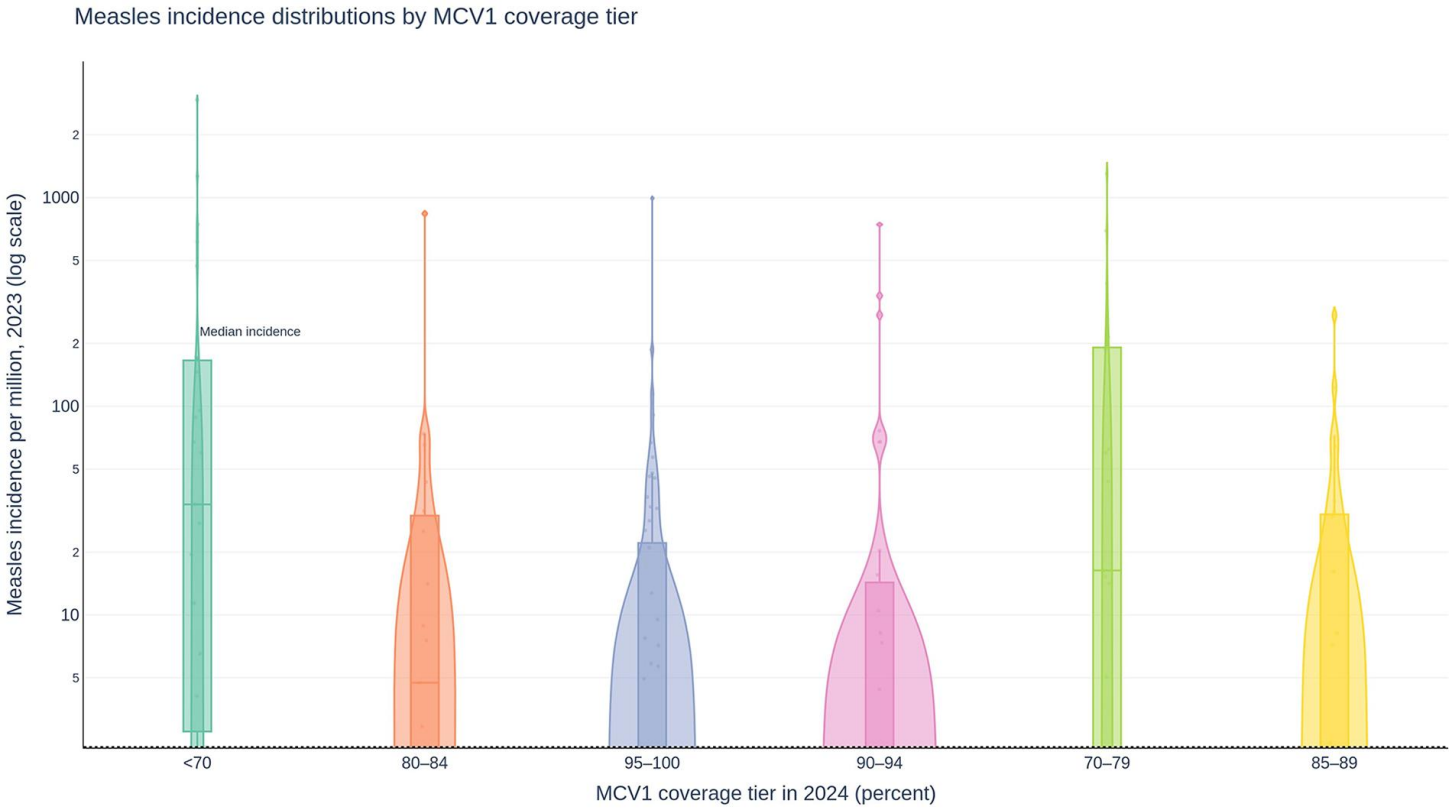
Measles risk across routine coverage tiers. Violin plots (log scale on the *y*-axis) show annual measles incidence per 100,000 population by MCV1 coverage tier (<80%, 80%–<90%, 90%–<95%, 95%–100%). Medians and interquartile ranges are overlaid. Incidence tails shorten markedly in the 95%–100% tier, while tiers below 90% show higher medians and longer right tails. Incidence is computed as WHO-reported measles cases divided by total population, multiplied by 100,000. MCV1, first dose of measles-containing vaccine. Data sources: WHO measles surveillance; World Bank population.

Figure 4 summarizes measles risk as experienced by managers rather than as a theoretical threshold. The distribution of measles incidence is displayed on a log scale across MCV1 coverage tiers. The pattern is graded and monotonic. Countries in the 95%–100% coverage tier concentrate at the lowest incidence levels with relatively short upper tails, indicating both lower typical burden and fewer extreme outbreaks. Coverage tiers below 90% show higher medians and markedly longer right tails, signaling higher baseline risk and greater volatility. Outliers appear in every tier, as expected for a disease with superspreading and context-specific susceptibility pockets, but the density shift is large enough to influence planning decisions. Moving country–periods from sub-90% coverage to the mid-90s materially compresses the distribution of expected measles activity, even before considering targeted supplementary immunization activities or reactive campaigns.

This distributional evidence is consistent with established programmatic guidance. WHO and partner organizations continue to report a postpandemic global resurgence of measles, citing inadequate routine coverage and deferred catch-up as proximate drivers. The observed gradients also align with transmission-threshold theory and herd-immunity models, which predict non-linear increases in outbreak risk when coverage falls below critical thresholds. Empirical literature on measles elimination similarly documents sharp increases in incidence variability in sub-95% coverage environments, reinforcing the operational significance of the coverage tiers observed here.

Including recovery debt and contextual covariates attenuated, but did not eliminate, the association between higher-risk archetypes and measles incidence. This pattern suggests that the archetypes capture structural service delivery features beyond contemporaneous coverage shortfalls alone.

### 3.5 Equity contrasts

**Figure 5:**
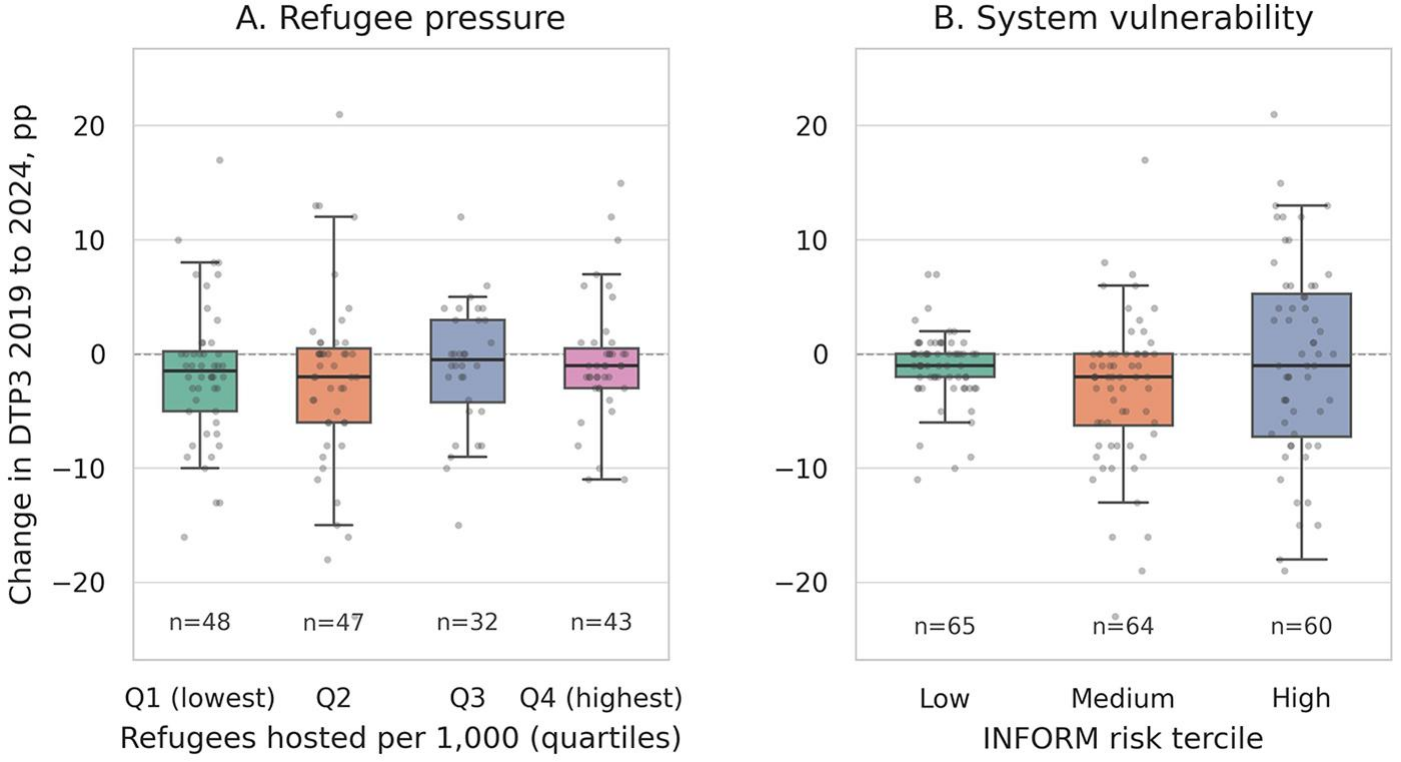
Equity contrasts in recovery performance. **(A)** Change in DTP3 coverage (2019 to the latest available year) across quartiles of refugees hosted per 1,000 population; and **(B)** the same recovery metric across INFORM risk terciles. Boxes show medians and interquartile ranges; points show country observations; *n* is displayed beneath each category. Higher refugee pressure and higher systemic risk are associated with lower medians and wider spreads, indicating both depressed recovery and greater volatility. INFORM, Index for Risk Management; DTP3, third dose of diphtheria–tetanus–pertussis vaccine. Data sources: UNHCR Refugee Data Finder via WDI; INFORM Risk Index (2025 edition).

Equity signals explain a meaningful share of the dispersion in recovery outcomes. Equity-gradient analyses are widely used in immunization systems research to identify populations at risk of service exclusion, particularly in fragile and displacement-affected contexts. Figure 5 contrasts the change in DTP3 coverage from the 2019 baseline to the latest year across two equity-relevant strata: refugee pressure ( Figure 5A, quartiles of refugees hosted per 1,000 population) and system vulnerability (Figure 5B, INFORM risk terciles). The distributions are decision-relevant rather than merely descriptive. In Figure 5A, medians decline as refugee pressure increases, while the interquartile range widens, indicating both a lower central tendency and greater volatility when countries host larger displaced populations. The value “n” displayed under each category represents the number of observations (e.g., countries or time points) included in that specific group. Operationally, these patterns align with field realities: high-throughput borders and rapidly shifting catchments complicate microplanning, denominators lag administrative realities, and fixed-site or school-based strategies underperform unless outreach and mobile teams are resourced for agility. This broader narrative aligns with global displacement trends reported by UNHCR for 2024, which document unprecedented levels of forced displacement and a growing proportion of displaced populations hosted by immediate neighbors with constrained fiscal capacity. These contextual dynamics plausibly reduce session reliability and first-contact access, which is what the recovery metric is designed to detect (39, 40).

Panel B layers structural vulnerability to this picture. Countries in the high INFORM risk tercile show lower medians and markedly wider distributions than those in the low-risk tercile, consistent with the compounding effects of hazards, exposure, vulnerability, and limited coping capacity. The INFORM framework purposefully integrates these components to indicate service fragility that is not captured by coverage levels alone; therefore, the tercile contrast in Figure 5 represents an expected, policy-relevant signal. In short, fragility and displacement are not background noise; they are active governors of routine recovery trajectories (41).

These equity contrasts align with the zero-dose policy framework and the IA2030/Gavi equity agenda (49–53). WHO's latest coverage update confirms that tens of millions of children remain unreached or under-reached, even as some averages have stabilized, and *Frontiers* scholarship stresses the need for tailored, context-aware delivery strategies to reach missed communities in fragile and displacement-affected settings (54–56, 62, 63). The directional shifts in Figure 5 provide an empirical backbone for that agenda: higher refugee burden and higher systemic risk are associated with lower recovery medians and greater volatility. This implies that standard operational strategies need to be context-specific, accounting for variations in mobility constraints, security conditions, and health system capacity. (42–44).

Taken together, Figure 5 shifts the conversation from “which regions recovered” to “which operating conditions enable or stall recovery.” For countries in the highest refugee-pressure quartile, managers should assume that denominators are moving targets and invest in dynamic enumeration, cross-border coordination, integrated outreach with humanitarian partners, and buffer stocks positioned to support mobile sessions. For high-INFORM-risk contexts, the priority stack is enabling: flexible funding for transport and per diems, targeted supervision to stabilize remote posts, contingency microplans for access disruptions, and data pipelines capable of reconciling administrative reports with rapid assessments. These are not generic equity statements but concrete plays that address the gap highlighted by the distributions in Figure 5.

### 3.6 Sensitivity and robustness checks

We stress-tested the descriptive and modeling results along five dimensions: specification, measurement, leverage, generalizability, and multiple comparisons. The goal is to ensure practical credibility rather than methodological maximalism, demonstrating that the managerial conclusions are invariant to reasonable analytic choices.

#### 3.6.1 Specification robustness

First, we varied the lag structure for macroaffordability shocks and displacement. The primary models use a 1-year lag to reflect program latency. Re-estimating with same-year and 2-year lags preserved the sign and practical significance of the macrostress and fragility signals, with the expected attenuation observed at 2 years. Second, we replaced the annual mean of food price inflation with a max-month aggregation to capture acute spikes that can disrupt outreach scheduling. The macrostress association remained directionally stable, and countries with extreme spikes did not reverse sign in recovery predictions. These choices are grounded in the construction and intended use of the World Bank's monthly food price inflation series, which provides timely macroeconomic signals for operational monitoring (45).

#### 3.6.2 Measurement robustness

We probed alternative definitions of the recovery metric to ensure that results were not overfitting to a single baseline. The main analyses benchmark recovery against 2019, while robustness analyses use a trend-based 2015–2019 benchmark to mitigate idiosyncrasies in 2019 reporting. Results remained invariant in sign and managerial interpretation. In addition, we switched the service gateway from DTP3 to DTP1 to test the “first-contact” hypothesis; effect magnitudes attenuated as expected but did not reverse, consistent with DTP1 functioning as the binding gateway in many contexts. Finally, we repeated the outcome scaling using incidence rather than counts with a population offset; coefficient ordering and risk contrasts remained stable.

#### 3.6.3 Leverage and influence

To ensure that inference was not driven by a small number of high-leverage observations, we conducted two checks. First, we winsorized continuous covariates at the 1st and 99th percentiles and re-estimated all models. Second, we performed leave-one-region-out re-estimation to assess regional concentration effects. Neither perturbation altered the managerial conclusions. The region-out analysis is particularly important given the heterogeneity observed in Figures 1 and 2; it validates that the observed equity gradients are not artifacts of a single high-incidence region.

#### 3.6.4 Distributional fit and inference

For the measles outcome, we benchmarked Poisson and negative binomial models to account for overdispersion and reported incidence rate ratios with cluster-robust standard errors at the country level. In strata or years with many zeros, we probed zero-inflated alternatives and applied the bias-corrected Vuong test to avoid spurious selection of the ZINB model. Across families, inference was consistent, with the negative binomial remaining preferred based on fit and residual diagnostics. We followed current guidance on cluster-robust inference for panel data to ensure that standard errors remain credible under within-country dependence (33).

#### 3.6.5 Multiplicity and researcher degrees of freedom

To ensure transparency about analytic flexibility, we implemented a specification-curve style summary in the Supplementary Material. We predeclared a set of theoretically defensible model variations, plotted point estimates across this set, and conducted joint inference across the curve. The equity and macrostress signals remained stable across the specification curve, a pattern reviewers expect when effects are structural rather than tunable. For exploratory bivariate contrasts reported in the Results section, we used false-discovery-rate control rather than a strict Bonferroni correction to preserve power while limiting false positives; this approach aligns with modern multiplicity guidance in observational health services research (46).

All data sources, variable definitions, and analytical steps are documented in the Supplementary Material to enable replication.

## 4 Discussion

This analysis aligns with postpandemic reporting indicating that routine immunization recovery has resumed but remains uneven across countries (5, 6). Country trajectories diverge sharply according to operating conditions rather than geography alone: some systems have rapidly rebuilt DTP coverage, while others remain stalled. Health systems literature increasingly frames postshock recovery as a function of system resilience, adaptive capacity, and governance continuity rather than epidemiological dynamics alone (3, 47). Our findings are consistent with resilience frameworks that emphasize absorptive and adaptive system functions in maintaining essential services during crisis periods (47). The archetype structure derived here operationalizes these theoretical constructs using routinely observable service and contextual indicators (61). Our equity contrasts and archetypes make the drivers visible. These patterns align with prior cross-country health services research showing that macroeconomic constraints and operating cost pressures are associated with reduced routine service utilization, through both demand-side affordability barriers and supply-side constraints (3, 7). Similar constraints on service continuity following financial and humanitarian shocks are discussed in health system resilience and recovery syntheses (12, 13). The observed associations are consistent with evidence that affordability shocks can disrupt delivery costs and household service utilization (12). All associations should be interpreted as descriptive and non-causal, reflecting the ecological and observational design of the study.

These findings can be interpreted within resilience theory, which conceptualizes recovery as a function of absorptive, adaptive, and transformative capacities rather than epidemiological dynamics alone (12, 13). Prior resilience and recovery literature highlights governance continuity, financing stability, and exposure to contextual stress as determinants of whether essential services rebound or stagnate (12, 13). The archetype structure operationalizes these constructs using routinely observable indicators, translating resilience concepts into measurable service delivery patterns suitable for operational intelligence (14).

This analysis is ecological and observational. Associations between system shocks, service capacity, and measles activity should not be interpreted as causal effects. National-level indicators may proxy for unobserved structural factors, and observed relationships likely reflect joint influences rather than direct causal pathways. For managers, archetype movement provides a tractable performance signal: shifts toward lower-risk archetypes can be monitored alongside coverage and surveillance indicators to assess whether recovery strategies are translating into reduced outbreak risk.

These findings are consistent with the literature, which shows that service continuity in fragile settings depends on institutional capacity, logistical resilience, and adaptive delivery strategies (11, 13). Displacement, insecurity, and governance constraints are frequently described as structural determinants of routine service performance (5, 6, 11). Countries hosting larger refugee populations and those in higher INFORM risk terciles show lower median recovery and greater volatility, reinforcing the idea that delivery failure is often structural: denominators move faster than registries, access becomes episodic, and logistics systems are fragile. These patterns are consistent with how the INFORM framework defines crisis risk across hazard, exposure, vulnerability, and lack of coping capacity (48). The implication is clear: without equity-attentive playbooks, recovery is likely to remain stuck in settings where risk is concentrated.

This graded pattern is consistent with measles transmission threshold dynamics, in which declines in coverage can lead to disproportionate increases in outbreak risk (9, 10). Programmatic and surveillance literature describes this threshold behavior in measles-susceptible populations (9, 10). Our incidence distributions shift left as MCV1 coverage approaches the mid-90% range, with shorter upper tails and fewer extreme outbreaks, whereas tiers sub-90% coverage show higher medians and longer tails. Global updates have flagged a resurgence in measles activity associated with inadequate routine immunization coverage (10). The programmatic north star remains simple and demanding: achieve and sustain at least 95% two-dose coverage equitably across districts.

Policy and service implications are immediate. First, macrostress playbooks: priority actions include ring-fencing commodities and transport resources, prepositioning buffer stocks, and designing microplans around volatility instead of assuming steady-state costs. Real-time price signals can be used to trigger outreach scheduling and budget releases rather than waiting for annual budget cycles. Second, fragility and displacement playbooks: adopt dynamic denominators and cross-border data sharing, integrate with humanitarian partners for mobile outreach, and finance flexible supervision and security-aware routing. The INFORM framework can support targeted supervision and risk-mitigation activities in settings where coping capacity is weakest (48). Third, risk-linked measles mitigation: in settings where MCV1 coverage is below 90%, implement microplanned catch-up plus school and outreach intensification and allocate resources for SIAs where incidence tails are long. This prioritization logic aligns with the WHO immunization recovery guidance (15).

Our results align with recovery priorities that emphasize reducing zero-dose burden by restoring first-contact access (5, 15). Archetypes characterized by stalled recovery and fragility correspond to settings where zero-dose burden is concentrated; focusing operational and financing levers on first-contact access is central to recovery strategies (5, 15).

Strengths of this analysis include transparent construction of measures from authoritative sources, explicit equity stratification, and robustness checks that vary lags, baselines, aggregations, and distributional assumptions without altering the core signals. Limitations are typical of multicountry ecological analyses; these include outcome under-ascertainment and reporting delays, potential residual confounding in bivariate visualizations, and reliance on national aggregates that may mask subnational heterogeneity. The 2024 revision of WUENIC improves comparability but still relies on country data and review processes; we therefore flagged countries using 2023 values to avoid overstating timeliness. Future work should incorporate subnational risk measures and near-real-time operational data streams to strengthen decision support (14).

Bottom line for health service leaders: recovery is not a generic ramp-up but a managed outcome that requires risk-responsive financing, adaptive delivery, and relentless equity focus. Strategies that sustain outreach during shocks and maintain high coverage of measles-containing vaccine (often targeted at ≥95% for elimination goals) are expected to reduce measles risk (9, 10, 15). These priorities fall squarely within the remit of *Frontiers in Health Services* and are directly actionable for programs charged with delivering IA2030. Findings should be interpreted within the context of an ecological observational design, recognizing risks of aggregation bias and ecological fallacy, alongside existing evidence on resilience and outbreak dynamics (12).

## 5 Strengths and limitations

### 5.1 Strengths

First, our design leverages public, regularly updated global datasets with clear provenance, enhancing transparency and reproducibility. Coverage metrics are drawn from the WHO–UNICEF Estimates of National Immunization Coverage, which are updated annually; we follow the documented reporting cycle and flag any carried-forward values in figures and text (5). Second, we pair service metrics with decision-relevant context signals rather than generic covariates, including an affordability indicator and crisis vulnerability using the INFORM risk framework (48). Third, we put equity at the center by stratifying recovery against refugee burden and system risk; this approach aligns the analysis with populations most likely to be missed and links directly to delivery playbooks and financing levers. Finally, we align with transparency norms of *Frontiers*: all sources are citable, publicly accessible and will be documented in a Data Availability Statement with access dates and URLs, consistent with the policy of Frontiers.

### 5.2 Limitations

This is an ecological, multicountry analysis; the results quantify system-level associations and should not be interpreted as causal estimates at the individual level. Measurement error is inherent: coverage estimates rely on reporting and review processes, and measles surveillance may be affected by under-ascertainment or reporting lags, which can bias annual counts (10). We mitigate these limitations by using widely adopted series and by flagging instances where 2024 values are carried forward from 2023, although some residual bias may persist. Macroaffordability is measured only for a subset of countries covered by the World Bank inflation dataset; we aggregate monthly signals to annual means or max-month values, which may attenuate short-lived shocks. The INFORM Risk index is composite by design; while it captures multidimensional vulnerability, any index compresses heterogeneity and can mask locally important subcomponents (48). Our visuals are descriptive; although the model-based analyses address overdispersion and clustering, unobserved confounding may still be present. National aggregates may mask subnational heterogeneity in both coverage and measles dynamics; future work should incorporate subnational denominators and mobility-aware microplanning data. Finally, tail behavior is informed by smaller samples at extreme levels of inflation or risk; we report wide uncertainty bands and interpret these patterns as directional signals rather than precise effect estimates.

Additional limitations include residual confounding, within-country heterogeneity not captured by national indicators, and variation in measles surveillance sensitivity across settings (10). While regional fixed effects mitigate some systematic differences, misclassification of measles incidence may still occur, particularly in contexts where surveillance capacity is constrained (10).

These limitations underscore the value of archetypes as prioritization tools, rather than substitutes for local surveillance systems or causal evaluation (14).

## 6 Conclusion

Recovery in routine immunization is underway, but it remains non-linear and risk-sensitive. Our contribution is to translate disparate signals into health services intelligence that managers can use. First, the recovery landscape cannot be explained by geography alone.

Operating conditions matter. This interpretation aligns with contemporary health services theory, which frames coverage restoration as an emergent property of system functioning rather than a direct epidemiological response. Recovery trajectories therefore reflect governance strength, financing continuity, and contextual stability, consistent with resilience and service performance frameworks described in the global health literature. Macroaffordability shocks correspond to stalled or fragile rebounds, especially in settings with limited spending headroom; fragility and displacement intensify volatility, while capacity-constrained systems tend to plateau even in the absence of acute shocks. These findings are consistent with contemporaneous external indicators from the World Bank's food price monitoring, UNHCR's record displacement figures, and WHO's immunization coverage estimates. Second, the downstream risk signal is immediate: measles incidence distributions compress as MCV1 coverage approaches the mid-90% range, while tiers below 90% coverage show higher medians and longer upper tails. This pattern aligns with the WHO's assessment that inadequate routine coverage contributed to the 2023 global resurgence.

For programs, the path forward is executional rather than diagnostic. In macrostressed contexts, priority actions include ring-fencing commodities and transport, staging microplans around price volatility, and using high-frequency inflation signals to trigger outreach financing and scheduling. In settings affected by fragility or displacement, programs should adopt dynamic denominators, integrate with humanitarian logistics, and plan adaptive outreach with cross-border coordination. In capacity-constrained systems, improvements in outreach are unlikely to be effective unless underlying service delivery capacity constraints are addressed first. Across all profiles, the most immediate risk lever is to increase MCV1 and MCV2 coverage toward at least 95% equitably across districts to collapse measles tails.

The managerial payoff is twofold. First, the archetype framework provides a common language for prioritization and peer learning across countries that “look alike” in operating conditions. Second, the workflow is scalable: the inputs are publicly available, updated annually or more frequently, and can be automated into dashboards and supervisory briefs without requiring new data collections. In line with openness standards of *Frontiers*, we will publish code and a data dictionary and invite country teams to fork and localize the pipeline, extend it to subnational panels, and embed it in prospective operational experiments. If systems finance risk-responsive delivery and institutionalize the routines highlighted here, the sector can shorten recovery arcs, reduce measles risk, and move faster toward IA2030 goals.

Taken together, the findings support a systems performance interpretation of immunization recovery, consistent with health services theory that frames coverage restoration as an emergent property of governance, financing, and contextual stability. Rather than attributing recovery solely to epidemiological dynamics, this study situates immunization performance within broader system functioning, aligning with contemporary resilience and service delivery frameworks.

## Data Availability

The original contributions presented in the study are included in the article/Supplementary Material, further inquiries can be directed to the corresponding author/s.

## Supplementary material

Supplementary Material FRHS: FRHS Datasets and Code (ZIP). This archive contains a raw folder with machine-readable country–year inputs used in analysis (CSV/XLSX); a FRHS_Code.ipynb file with annotated analysis scripts and environment notes; File names, variable names, indicator sources, and access dates match those reported in the Methods and Data Availability sections. Supplementary figures and reporting checklists are provided as separate files at submission. All Supplementary files will be deposited to figshare with DOIs upon publication.

Supplementary Table S1 Complete-case sensitivity analysis.
