# Supplementary material for: Operational intelligence for immunization recovery: mapping system shocks and capacity to zero-dose debt and outbreak risk
Source: Front Health Serv. 2026 May 7;6:1745633. doi: 10.3389/frhs.2026.1745633 (PMC13190393; doi:10.3389/frhs.2026.1745633)
Supplement: Supplementary Table S1 — Complete-case sensitivity analysis. [file Table1.docx]

**Table S1**. Complete-case sensitivity analysis

| **Predictor** | **Main analysis IRR** | **Complete-case IRR** | **Directionally consistent** |
| --- | --- | --- | --- |
| Archetype B vs A | 1.20 | 1.18 | Yes |
| Archetype C vs A | 1.55 | 1.52 | Yes |
| Archetype D vs A | 2.10 | 2.05 | Yes |
| Zero-dose debt (DTP1) | 1.03 | 1.03 | Yes |
